# Participants’ perceptions of a knowledge-brokering strategy to facilitate evidence-informed policy-making in Fiji

**DOI:** 10.1186/1471-2458-13-725

**Published:** 2013-08-07

**Authors:** Gade Waqa, Helen Mavoa, Wendy Snowdon, Marj Moodie, Rigieta Nadakuitavuki, Marita Mc Cabe, Boyd Swinburn

**Affiliations:** 1 Pacific Research Centre for the Prevention of Obesity and Non-Communicable Diseases (C-POND), Fiji School of Medicine, College of Medicine, Nursing and Health Sciences, Fiji National University, Suva, Fiji; 2 WHO Collaborating Centre for Obesity Prevention, Deakin University, Melbourne, Australia; 3 Deakin Health Economics, Deakin University, Melbourne, Australia; 4 Ministry of Health, Suva, Fiji; 5 School of Psychology, Deakin University, Melbourne, Australia; 6 School of Population Health, University of Auckland, Auckland, New Zealand

**Keywords:** Knowledge-brokering strategies, Knowledge exchange, Evidence-informed policy-making, Perceived barriers

## Abstract

**Background:**

Evidence-informed policy-making (EIPM) is optimal when evidence-producers (researchers) and policy developers work collaboratively to ensure the production and use of the best available evidence. This paper examined participants’ perceptions of knowledge-brokering strategies used in the TROPIC (Translational Research in Obesity Prevention in Communities) project to facilitate the use of obesity-related evidence in policy development in Fiji.

**Method:**

Knowledge-brokers delivered a 12-18 month programme comprising workshops targeting EIPM skills and practical support for developing evidence-informed policy briefs to reduce obesity. The programme was tailored to each of the six participating organizations. Knowledge-brokering strategies included negotiating topics that were aligned to the goals of individual organizations, monitoring and evaluating time-management skills, accommodating other organizational and individual priorities, delivering practical sessions on use of appropriate research tools and supporting individual writing of policy briefs. Two qualitative methods were used to examine individuals’ perceptions of skills obtained, opportunities afforded by the TROPIC project, facilitators and inhibiters to planned policy brief development and suggestions for improved programme delivery. Forty-nine participants completed an electronic word table and then participated in a semi-structured interview. An independent interviewer conducted structured interviews with a high-ranking officer in each organization to examine their perceptions of TROPIC engagement strategies. Data were analyzed descriptively and thematically, with the first author and another experienced qualitative researcher analyzing data sets separately, and then combining analyses.

**Results:**

Many participants believed that they had increased their skills in acquiring, assessing, adapting and applying evidence, writing policy briefs and presenting evidence-based arguments to higher levels. Many participants preferred one-to-one meetings to group activities to ensure early resolution of developing issues and to refine policy briefs. Perceived barriers to EIPM were lack of knowledge about data sources, inadequate time to develop evidence-informed briefs, and insufficient resources for accessing and managing evidence.

**Conclusion:**

An innovative knowledge-brokering approach utilizing skill development and mentorship facilitated individual EIPM skills and policy brief development. The TROPIC model could stimulate evidence-based policy action relating to obesity prevention and other policy areas in other Pacific countries and elsewhere.

## Background

Over the past few decades, overweight and obesity have become a major public health issue both globally [1,2] and among Pacific Island nations [3-5] including Fiji [6-8]. A worrying trend is that the high prevalence of childhood obesity in most Pacific Island communities [9,10] has more than doubled [11]. The concept that children would outgrow overweight and obesity as they developed has not been supported by evidence, with the tracking of increased adiposity from childhood into adulthood [12-14]. Since obesity is increasingly associated with significant health problems in younger age groups and is an important risk factor associated with adult morbidity and mortality [15,16], the trend toward increased obesity prevalence must be reversed [17]. Despite strong evidence supporting the efficacy of health promoting approaches to reduce obesity among younger children [18-20], it appears that such approaches, while probably necessary, are not alone sufficient to overcome significant economic, physical and sociocultural barriers to sustaining a healthy weight [5,21,22]. Policies, laws and regulations are needed to drive environmental and social changes that will eventually have a sustainable impact on reducing obesity [23].

Promising models of integrated policy interventions that support healthy diets and physical activity and could potentially improve environmental factors have been widely recognized [24], but not widely implemented [25-27]. Despite the need for policy action to create healthier environments, little is known about policy approaches that are most effective in preventing obesity [28-30]. More policies are needed to improve food and physical activity environments, especially policies from outside the health sector [31]. Public health practitioners are key to implementing and evaluating public policies that impact on health [32,33]. However, the engagement of sectors outside health, particularly education, transport planning and agriculture, will be important to the long-term success of policy changes towards obesity prevention [23]. All key players in obesity prevention (governments, international organizations, the private sector and civil society/non-governmental organizations), need to take a leadership role and drive policy changes [34-36]. Despite the known importance of these required changes, Swinburn [17] found that many government policies such as the banning of junk food marketing to children have encountered heavy opposition from the corporate sector [37]. There is a great need, therefore, to better understand the decision-making processes of policy-makers in order to develop more effective evidence-based approaches to policy development [38-40].

Research can play a variety of roles in policy formulation. Without evidence, policy-makers fall back on perception, ideology, or conventional wisdom, and many policy decisions have indeed been made on this basis [40]. Emphasis has recently been placed on the need for more “evidence-based” or “evidence-informed” policy-making to help solve complex public policy problems [41]. Recent studies by Nutley *et al*[42] and Edwards [41] confirmed that the impact of research is greater when it is part of policy development and decision-making processes. However, the utilization of research evidence in policy development remains challenging, with large gaps between research and policy-makers [41,43,44]. In recent years, knowledge-brokers have become key players in bridging the gap between evidence producers and evidence users [45,46] by increasing both awareness and use of the best available evidence to inform policy [47] and/or practice [48], as well as to facilitate the dissemination of relevant evidence to policy makers. Knowledge brokering refers to promoting interaction between researchers and end-users of evidence [47]. We elected to draw on the concept of knowledge exchange because we were employing strategies to promote interaction between producers and users of knowledge [30,49] and were taking of the role of being a “linkage agent”[39,41] within and between participating organizations.

Despite the diverse challenges in developing evidence-informed policy making in resource-poor countries, some challenges of introducing evidence-based policy approaches are common to all settings with limited resources: barriers to use of evidence, widespread underfunding, insufficient human resources, lack of incentives or capacity to draw evidence, limited access to technology and inadequate information for decision making.

This paper focuses on the Translational Research on Obesity Prevention in Communities (TROPIC) project, a natural extension of the Pacific Obesity Prevention in Communities (OPIC) project that generated substantial data on adolescent obesity through the delivery of multi-faceted interventions in school and community settings in Fiji, Tonga, New Zealand and Australia. The TROPIC project investigated the effect of knowledge-brokering approaches on the uptake of evidence from OPIC and other sources to inform obesity-related policy in six organizations in Fiji [50]. In line with Lavis *et al*[39], one of the main targeted outcomes of TROPIC was to utilize research evidence in the development of policy briefs, leading to more effective policy decisions and practices and, subsequently, improved health outcomes. Evidence-informed decision-making (EIDM) involves the translation of the best available research evidence to inform policies, programs and practices [51], making it appropriate for both government and non-government sectors. One way to increase the use of evidence in policy is to employ a knowledge-brokering approach to bridge the gap between researchers and evidence users [45,52,53]. In this study, the primary objective of the knowledge-brokering team was to exchange information with participants and participating organisations. Participants provided information on strategic planning and policy cycles and negotiated topics for policy briefs. Using a knowledge exchange model [54], the KB team provided skills for EIPM, relevant evidence and supported the development and presentation of policy briefs, as well as keeping focal points informed of progress and challenges to developing policy briefs. The research question for the TROPIC project was: Can a knowledge-brokering approach advance evidence-informed policy development to improve eating and physical activity environments in Fiji? This paper explores the perceptions of 55 participants (49 participants; 6 high-level officers) involved in the TROPIC project about the knowledge-brokering approach that was used to develop evidence-based policies that had the potential to reduce obesity in Fiji.

## Methods

### Project structure

TROPIC was a three year (June 2009 to October 2012) project funded by an AusAID Australian Development Research Award grant. The project was conducted by Deakin University in collaboration with the Fiji National University [50]. TROPIC was approved by the Deakin University Human Research Ethics Committee, the Fiji Health Research Committee and the Fiji National Research Ethics Review Committee. Details of the knowledge-brokering process employed in TROPIC have been reported separately in Waqa *et al* 2012 (submitted). In brief, the process comprised workshops and individualized support to develop policy briefs.

### Study sample

A purposive sample of four government and two non-government organizations with potential to create or positively influence policies to improve food and/or physical activity environments were recruited [50]. Government organizations directly and indirectly involved in health were recruited because of their potential to influence policies that could impact positively on health. Swinburn [23] notes that the influence of non-health sectors on health-related policies is often greater than that of Ministries of Health. Focal points who were senior staff members (contact person) in each participating organization identified and recruited participants with either policy-making or advocacy roles. Forty-nine junior, middle and senior managers participated in the study from these organizations, however, only 63% fully participated in both workshops and policy brief development while the rest cited heavy work-loads, taking up postgraduate scholarships, resignation from their post, or relocation either within Fiji or overseas as reasons for failing to complete the project. Therefore, the data collected was only from this 63% of the initial participants recruited.

A high ranking officer from each of organization and who was familiar with TROPIC took part in a structured interview.

### Data collection and analysis

An electronic word table (Table 1) was developed and piloted for clarity with four individuals who were familiar with TROPIC knowledge exchange strategies. The questions used in the electronic table sought to examine individuals’ perceptions of skills obtained during TROPIC, opportunities afforded by the project, aspects that could be improved, and facilitators and inhibiters to planned policy brief development. On completion of the TROPIC project and submission of policy briefs, the electronic word table was emailed to all participants to complete prior to taking part in a short face-to-face interview.

**Table 1:** Electronic word table questionnaire

**Participant ID:**		**Date:**
**Question**	**Response**	**Example**
Briefly describe up to **five** skills that you have gained personally from TROPIC. Give a specific example of each skill		
Briefly describe up to **five** opportunities that you have had/will have to further your career as a result of being in TROPIC.		Not required
List the three best things about TROPIC		Not required
List three things about TROPIC that could be improved		Not required
List things that have made it easy to complete policy briefs (if you did)		
List things that have made it hard to complete policy briefs		

Thank you and vinaka vakalevu for completing this word table.

In examining participants’ perceptions of the knowledge-brokering strategies used in TROPIC, one-to-one semi-structured interviews were conducted to determine 1) the skills and opportunities gained by participants; 2) the challenges that participants made faced in preparing policy briefs, and 3) the effectiveness of knowledge-brokering engagement strategies employed in the project. These interviews sought to gain clarification and, where necessary, to obtain further detail from individual participants in relation to the overall research question.

Prior to the interviews, the electronic word table and the pre—and post-TROPIC surveys that were completed by each participant were reviewed by the knowledge-brokering team. Given that interviews were conducted by a member of the TROPIC team, steps were taken to ensure interviewees felt comfortable sharing negative as well as positive perceptions of the programme. This included an emphasis on anonymity and confidentiality and the need for feedback to inform future activities. An individualized interview guide was developed to fill in any data gaps and to elaborate on responses from the pre-and post-TROPIC survey [50] and the word table, and to provide an opportunity for participants to raise any additional relevant issues.

In addition, a high-ranking officer (e.g. Permanent Secretary or the Minister) from each of the six organizations, identified prospectively by the research team, was invited to participate in a structured interview of around 45 minutes duration in order to examine their perceptions of the engagement processes that were used in TROPIC. More specifically, these interviews were designed to gauge awareness and expectations of the project, organizational engagement with TROPIC, perceived impacts on the organization and plans to sustain evidence-informed decision-making processes following TROPIC. To ensure impartiality, these interviews were conducted by an independent external consultant.

Semi-structured and structured interviews with both participants and high-level officers respectively were conducted in English at a time and place convenient to each participant, and were of an average duration of 45 minutes. The digital records of interviews were transcribed and managed on N-Vivo 8.0 software for the analyses of qualitative data (QSR International, Melbourne, Australia).

Data were analyzed using content and thematic analyses, with the first and second authors analyzing the participant’s data separately and then combining analyses. In line with other studies, like Kothari et al., data were analyzed through a combination of content and thematic analyses [54]. Content from the participant interviews was identified by the first and second authors independently, then together with agreement on each item that was coded into N-vivo 8.0. Subsequently, the data were analyzed for themes that emerged inductively. The high-level interviews were analyzed by another qualitative expert and the first author using a similar approach to the one described for the participant interviews. The interview and electronic word table collected different data as described. The data from the two sources were analyzed separately and responses were not matched to participants.

## Results

### Participants

All participants completed and returned the electronic word table and participated in the subsequent interview which was conducted by a member of the knowledge-brokering team. One of the six high-ranking officers refused to be interviewed citing security concerns. The participants’ perceptions of knowledge-brokering strategies are discussed under the following themes: 1) skills gained and opportunities afforded; 2) challenges in completing policy briefs; and 3) the effectiveness of the different engagement strategies used.

### Skills gained and opportunities afforded

The first part of the electronic word table examined the skills and opportunities participants had gained during TROPIC. Many participants indicated that they had increased their skills in acquiring, accessing, adapting and applying evidence. All participants gave examples of specific analytical strategies, including how and where to source evidence, how to use groups to effectively support learning as in critical appraising other policy briefs, and providing feedback on a series of policy drafts. One of the respondents said:

*I think in the civil service, we don’t really have a lot of people that are policy inclined in terms of writing skills. It’s something that has to be developed, people could have passion but they might not have the abilities … it sure justifies the reason to have a lot more people involved* [in TROPIC] *due to the skills that have been acquired from such a great package.*

Many participants felt that the reports or discussion papers that they had previously prepared were unsound because they focused primarily on perceptions and anecdotal evidence and seldom drew on evidence. Some participants, particularly those who had searched and used sound evidence, that is, evidence derived from reputable studies or surveys that are relevant for the Pacific to support their policy brief arguments, felt that TROPIC was a good learning opportunity:

*….it’s like an eye opener…, so it has really instilled a passion to search* [for evidence]…*what message to give out to change the behavior …I look at the literature to support that, so it takes long*er *to actually come out with things but it’s a good learning experience.*

Some high-level decision-makers from government organizations felt that TROPIC provided more opportunities to collaborate and improve networking with other partner organizations. However representatives from the non-government organizations differed in that advocacy and networking has always been part of their organization’s core business. An interviewee from a relatively small organization commented that they could only send one staff member, thus limiting the capacity building opportunity that TROPIC afforded. Another interviewee reported that while they had pre-existing links with organizations that engaged in policy and/or programme development to increase healthy nutrition and physical activity, the opportunities for networking and collaborative policy development provided by TROPIC had strengthened those relationships. Some participants, in particular high-level decision makers, acknowledged the importance of having good research and communication skill. Other participants noted the potential to network with other experts in advocating for policy intervention in the commercial market or public sectors in order to achieve better health.

*.. I am required to have good research skills as well as advocacy skills. The skills I learnt in TROPIC have helped me enhance my research skills and also look at ways of linking and backing advocacy with credible research to make it more effective and most of all how to draft good policy papers. All these* [skills] *will and have definitely helped me enhance my professional skills.*

The improved skills in accessing and utilizing evidence as well as in the writing of policy briefs were seen to be connected not only to the writing of policy briefs, reports and academic papers, but also to committees outside their workplace whom they served.

*The skills that I learned are not only utilized in the workplace; I have written a few other papers apart from the two policy briefs that has helped me ….and ….also in our community, I was able to write up a few papers on financial procedures for running a school and other policy papers that …. will guide those who are involved in the running of the schools where I am a committee member. The skills and knowledge were acquired from this TROPIC workshop* [initiative] *and I’m very thankful to that.*

One of the advantages of TROPIC is that participants were able to present evidence-based arguments to higher-level officers. This required a high level of written and presentation skills.

TROPIC gave me an opportunity to market my policy topics to the intended audience, meaning that it was the first time to present such policy briefs to a high level authority.

About 45% of participants attended at least one workshop, but did not complete the 12-18 months intervention, citing the following reasons for non-completion: heavy work-loads, taking up postgraduate scholarships, resignation from their post, and in the case of a number of participants, relocation either within different sectors in Fiji or overseas. A fellow participant described this as a “missed opportunity”:

This program has been a *missed opportunity for most of our staff* [that did not complete the package]*. As they move up the hierarchy they will tend to focus more on management and …part of it is about reviewing or maybe developing a new policy, so it is a missed opportunity for them.*

#### Challenges of completing policy briefs

Whilst the majority of participants believed that they had gained multiple skills on evidence use from TROPIC, many of them felt that they had limited opportunities in accessing evidence due to lack of access to internet or libraries. Some participants felt that they had insufficient access to either full papers (vs. abstracts) or papers from high quality journals.

….*we* [organization] *need access* [to library and internet] *and in particular to the good literature that requires us to pay ….and we cannot afford to subscribe to every different journal.*

Some participants described the difficulty in accessing information either from different departments within their organisation or from other organizations because of the different policy making systems. Because of limited networking opportunities, many participants tended to draw on the information they had immediate access to rather than approaching other organizations:

Certain *challenges exist* within different departments in the same organization *and other organizations like they expect us to* fill the form *describing the information needed* and [we] expect to hear back after a number of days. [Now] I *am thankful with the networking that TROPIC started as we meet and* [know] *the people that we* [usually struggled] *to see within the Ministry and those outside the Ministry and is not a challenge any more.*

The World Health Organization program provides health-related literature [55]. However, some participants believed that there is overall lack of access to research tools and good evidence:

There is overall lack of access to research tools*. The unavailability of good journals or access to HINARI* [WHO database] *and other research articles* [*fees*]*, lack of studies or research carried out in Fiji, unavailability of local data. Lastly far away distance – in isolation and difficult to contact or liaise with TROPIC from the western division, at times there’s problem with the network and getting access to a computer or internet.*

Many participants stated that they had limited time for accessing and utilizing evidence including local evidence from health divisions:

*I am very passionate about research and while I understand the importance of research and the impact it can have, that evidence-based research will help me draw up better cabinet papers, but one of the challenges is the time. The time factor* on *how many, how much we do in a week, how much we get to do the research in order to enhance our work, and in terms of accessibility to the database and latest statistics. We have challenges in that, sometimes our work colleagues in the division don’t realize how important databases are and in fact most of our work comes from grassroots level and they need to be providing us with updated information from the divisions, how well they communicate with our staff at headquarters in terms of ensuring that the database is updated on a timely manner….*

### Effectiveness of engagement strategies

Many participants acknowledged the importance of flexibility in the TROPIC initiative and explained how it influenced their learning:

[We have] *certain time frames set by the* [TROPIC] team *that we need to meet. The flexibility of the program and the team made it possible for us to achieve the target we set despite our or their busy schedules and work programs that clashes with our meeting time …*

The various forms of motivating strategies employed by the TROPIC knowledge-brokering team included both face-to-face group workshops and group meetings, both of which focused on critical appraisal of other policy briefs. However, the one to one meetings were the preferred method for most participants. Many participants commented on the reliability of the TROPIC team:

[The] motivating strategies have been good *……… when we are stuck in our policy writing….., they always come in and assist… and make sure that we keep on moving…*

Participants generally reported that the TROPIC workshops offered a supportive learning environment:

The learning environment was relaxed and provided an avenue for easy understanding of issues. This is important for adult learning because as adults our cognitive skills become slower and we need to learn in relaxed environment rather than classroom style teaching.

The practical sessions used during the workshop and one-to-one mentoring also encouraged learning, as the following quote illustrates:

The best thing about TROPIC was the emphasis on practical work with theory rather than being only based on theory. We were taught the research skills and then were told to identify a problem and write a policy paper. This was great because people learnt by doing things practically and also could easily relate to what they were doing.

For some, it is stepping stone to further their studies and career path:

*The knowledge and skills learnt* [from TROPIC] *helped me to improve my Masters Research project and career option as a Policy Writer.*

## Discussion

Important insights into skills gained, opportunities for, and barriers to, knowledge exchange engagement strategies were gained from a sample of decision makers in four government and two non-governmental organizations, as well as one high-level officer from each participating organization. The study findings identified a number of positive outcomes from the engagement processes used in knowledge-brokering activities in TROPIC as the approach was quite different from other knowledge-brokering approaches that have been described [45,49,56]. In addition, while organisational barriers limited the impact of using evidence in the development of policy or advocacy statements, the challenge was to introduce appropriate research tools that supported the use of sound and relevant evidence as highlighted by others [40,43].

Participants felt that the strategies employed by TROPIC enabled them to learn skills and gain knowledge about accessing, analyzing and using sound evidence for decision and/or policy-making that extended beyond what they had acquired simply through their own experiences as managers.

Participants also felt more confident in their potential roles as future policy writers. Additionally, participants suggested that, other job opportunities may open up given these new skills. Researchers often indicate a desire to share evidence effectively with policy-makers while simultaneously expressing concern about the skill gap between researchers and policy makers [47]. It is anticipated that the participants who successfully completed the TROPIC project will continue their careers with improved writing and searching skills and as a result of TROPIC, are more equipped to write evidence-informed policy briefs in their respective organizations. Participants believed that the knowledge-brokering practical approach tailored to mentorship and skill development facilitated individual learning. One of the significant developments during TROPIC was the development of a policy to promote a healthy work environment in three of the six participating organizations. One government department had a low completion rate that could be directly attributable to the need to divert participants to other essential roles following a hurricane.

There appeared to be some problems with practices that supported learning. Participants perceived the acquired research skills as an intellectually interesting process of exploring what works in other countries and how this could be adapted locally. For some, it was a stepping stone to further studies. Whilst some participants understood the engagement and learning processes, including the different approaches that enhance learning, most participants felt that it would be hard to practice the skills learned without having proper research tools and adequate time to utilize these new skills. This was in line with findings from other studies [40,47]. Furthermore, a number of participants acknowledged having greater respect for evidence-informed documents. The uptake of research findings into policy could be improved in low and middle-income countries through multi-faceted and tailored interventions to increase capacity and deal with resource constraints [57]. Much of the value of this research derives from its contribution to a very limited literature on the relationship between evidence and policy in resource-poor settings, particularly the South Pacific. This region faces significant challenges or impediments to help build the skills and knowledge required to lead and drive the use of evidence in policy-making [23,58,59].

The Fiji government, like their counterparts in many low- and middle-income countries, has limited economic and human resources with low access to technology and inadequate evidence for sound decision making. Nevertheless, the current Fiji government focused on evidence-based and performance-based achievements. Additionally, significant staff changes in the public service have limited the development of a critical mass of staff with EIPM skills.

This study is unique in that it is first to study the perceptions of participants about a knowledge-brokering approach designed to reduce obesity in a lower-middle income country in the South Pacific. It is also unique in that the knowledge-brokering team had in-depth local knowledge and was able to identify knowledge-brokering components that were likely to be effective in the specific context of Fiji.

There are some limitations of this study. There were insufficient NGOs and participant numbers within NGOs to make a valid comparison between NGO and government organizations. There was lack of control over selecting of participants resulting in participants with a wide range of EIPM skills and roles. Additionally, having TROPIC team members interview the participants had both strengths (interviewers had reviewed all data from each participant (baseline and follow up) prior to interview and limitations (potential for bias). Given the close relationships developed with the TROPIC team and the participants, who spoke freely during the TROPIC engagement process and made a number of suggestions for change, and that the interview transcripts included negative comments, it is unlikely that participants felt constrained in their responses. Care was taken to select the team member who had least involvement with each participant to conduct the interviews.

## Conclusion

The use of sound relevant evidence in decision- and/or policy-making has been widely researched and has been declared a major priority in policy and practice settings worldwide. Key attributes required of knowledge-brokers are excellent communication and motivational skills and the ability to facilitate interactions between evidence-producers and evidence-users. Participants who were time-poor indicated that they valued flexibility in programming, working on a policy brief as an exercise to reinforce evidence-informed policy-making skills and preferred one-to-one mentoring rather than small group activities. Whilst the findings of this study may not be generalizable to all knowledge-brokering contexts, they provide important understandings on the knowledge-brokering strategies preferred by time- and resource-poor policy-makers in a low-to-middle income country that is undergoing rapid policy reform. More analysis of knowledge-brokering activities that are appropriate to further develop evidence-informed policy development in Pacific and other low-to-middle income countries is necessary before this current innovative approach is used as a model to stimulate policy action in other Pacific countries.

## Competing interests

The authors declare that they have no competing interests.

## Authors’ contributions

GW conceptualized the paper and wrote the first draft and the final version. HM co-designed the project and co-analyzed the data, as well as critically reviewing all drafts. All authors critically reviewed the manuscript and approved the final version.

## Pre-publication history

The pre-publication history for this paper can be accessed here:

http://www.biomedcentral.com/1471-2458/13/725/prepub
